# Differential Expression of *ID4* and Its Association with *TP53* Mutation, *SOX2*, *SOX4* and *OCT-4* Expression Levels

**DOI:** 10.1371/journal.pone.0061605

**Published:** 2013-04-16

**Authors:** Thais Fernanda de Almeida Galatro, Miyuki Uno, Sueli Mieko Oba-Shinjo, Antonio Nogueira Almeida, Manoel J. Teixeira, Sérgio Rosemberg, Suely Kazue N. Marie

**Affiliations:** 1 Department of Neurology, School of Medicine, University of São Paulo, São Paulo, São Paulo, Brazil; 2 Center of Translational Oncology, Instituto do Câncer do Estado de São Paulo (ICESP), São Paulo, São Paulo, Brazil; 3 Department of Pathology, School of Medicine, University of São Paulo, São Paulo, São Paulo, Brazil; University of Navarra, Spain

## Abstract

Inhibitor of DNA Binding 4 (ID4) is a member of the helix-loop-helix ID family of transcription factors, mostly present in the central nervous system during embryonic development, that has been associated with *TP53* mutation and activation of *SOX2*. Along with other transcription factors, *ID4* has been implicated in the tumorigenic process of astrocytomas, contributing to cell dedifferentiation, proliferation and chemoresistance. In this study, we aimed to characterize the *ID4* expression pattern in human diffusely infiltrative astrocytomas of World Health Organization (WHO) grades II to IV of malignancy (AGII-AGIV); to correlate its expression level to that of *SOX2*, *SOX4*, *OCT-4* and *NANOG*, along with *TP53* mutational status; and to correlate the results with the clinical end-point of overall survival among glioblastoma patients. Quantitative real time PCR (qRT-PCR) was performed in 130 samples of astrocytomas for relative expression, showing up-regulation of all transcription factors in tumor cases. Positive correlation was found when comparing *ID4* relative expression of infiltrative astrocytomas with *SOX2* (*r* = 0.50; *p*<0.005), *SOX4* (*r* = 0.43; *p*<0.005) and *OCT-4* (*r* = 0.39; *p*<0.05). The results from *TP53* coding exon analysis allowed comparisons between wild-type and mutated status only in AGII cases, demonstrating significantly higher levels of *ID4*, *SOX2* and *SOX4* in mutated cases (*p*<0.05). This pattern was maintained in secondary GBM and further confirmed by immunohistochemistry, suggesting a role for *ID4*, *SOX2* and *SOX4* in early astrocytoma tumorigenesis. Combined hyperexpression of *ID4*, *SOX4* and *OCT-4* conferred a much lower (6 months) median survival than did hypoexpression (18 months). Because both ID4 alone and a complex of SOX4 and OCT-4 activate *SOX2* transcription, it is possible that multiple activation of *SOX2* impair the prognosis of GBM patients. These observational results of associated expression of *ID4* with *SOX4* and *OCT-4* may be used as a predictive factor of prognosis upon further confirmation in a larger GBM series.

## Introduction

Inhibitor of DNA Binding (ID) proteins (ID1–4) belong to the helix-loop-helix (HLH) superfamily of transcription factors and exert their functions through the highly conserved HLH dimerization domain. Due to the lack of a DNA binding domain, IDs sequester and inhibit the activity of their specific target proteins, playing important roles in cell cycle control, growth, differentiation, angiogenesis and tumorigenesis [1]–[4]. In healthy organisms, *ID* expression is up-regulated in stem and progenitor cells, maintaining self-renewal capacity, pluripotency and an undifferentiated state. However, *ID* expression declines to basal values when cells differentiate towards the destined specific lineage [5], [6]. The expression of ID1–3 proteins is widespread, while the ID4 expression pattern is restricted to the developing brain, particularly in neural progenitor cells [7]. The overexpression of IDs in tumor cells has been suggested to induce reversion to an embryonic-like state, with high rates of proliferation, migration and neo-angiogenesis facilitating tumor formation [4].

Astrocytomas are the most common primary brain tumors. World Health Organization (WHO) classifies the astrocytomas into four grades: grade I or pilocytic astrocytoma, grade II, or low-grade astrocytoma (AGII), grade III, or anaplastic astrocytoma (AGIII) and grade IV astrocytoma or glioblastoma (AGIV or GBM) [8]. Diffusely infiltrative astrocytomas (AGII-GBM) invade the surrounding normal brain tissue, hampering tumor resection. GBM is the most malignant and frequent brain tumor in adults and they can be divided into two subgroups: primary GBM, which arise de novo, and secondary GBM, which results from the progression of a lower grade astrocytoma [9], [10]. The malignant transformation of astrocytomas, is associated with augmented ID expression [3], particularly ID4 [11], [12]. Interestingly, the up-regulation of *ID4* has been associated with *TP53* mutation status [13], [14], which is an early event in astrocytoma progression; additionally, *TP53* mutation is more related to secondary GBM [9]. Moreover, hyperexpression of *ID4* was found to be a key regulator of malignant transformation of *Ink4a/Arf ^−/−^* (cyclin-dependent kinase inhibitor 2A, isoform 4) murine astrocytes in *in vivo* experiments, resulting in formation of high grade gliomas according to clinical and histological analysis [15]. These results may be consistent with astrocyte dedifferentiation to an immature progenitor-like state. It has also been demonstrated that ID4 protein activates SRY (sex determining region Y)-box 2 (*SOX2)* transcription in GBM and glioma stem cells [16]. Similarly, SOX4 and POU class 5 homeobox 1 (OCT-4) proteins were also shown to activate *SOX2* transcription in glioma initiating cells [17], [18]. Along with Nanog homeobox (*NANOG*), these transcription factors are highly expressed in embryonic, progenitor, and tumor stem cells, in contrast to the low levels of expression that are found in differentiated cells [19]–[21].

This study aimed to characterize the *ID4* expression pattern in human astrocytomas of grades II to IV of malignancy; to correlate its expression level to that of *SOX2*, *SOX4*, *OCT-4* and *NANOG*, along with *TP53* mutational status; and to correlate the results with the clinical end-point of overall survival among GBM patients. In parallel, expression of the neural and brain tumor stem cell marker *CD133* was assessed to better evaluate the progenitor cell condition [22]–[23].

## Materials and Methods

### Tissue Samples and Ethical Statement

One hundred and thirty diffusely infiltrative astrocytomas (grades II to IV) were obtained during therapeutic surgery of patients treated by the Neurosurgery Group of the Department of Neurology at Hospital das Clínicas at the School of Medicine of the University of São Paulo, in the period of 2000 to 2007. The cases were categorized according to the WHO grading system [8] by neuropathologists from the Division of Pathological Anatomy of the same institution. The studied series consisted of 26 AGII, 18 AGIII, 86 GBM, and 22 non-neoplastic (NN) brain anonymized cases from epilepsy patients subjected to temporal lobectomy. Demographic data of the studied cases is presented in Table 1, and the clinical findings are presented in Table S1. Samples were macrodissected and immediately snap-frozen in liquid nitrogen upon surgical removal. A 4µm-thick cryosection of each sample was analyzed under a light microscope after hematoxylin-eosin staining for assessment of necrotic, cellular debris and non-neoplastic areas (in tumor samples), followed by removal from the frozen block by microdissection prior to DNA and RNA extractions [24], [25]. Eighty-one GBM patients (94.2%) presented with onset of clinical symptoms within 3 months prior to diagnostic surgical intervention and were classified as presenting primary GBM. Five GBM patients (5.8%) presented a tumor which was resected over one year after a lower grade astrocytoma (grade II or III), and were designated as secondary GBM cases. Written informed consent was obtained from all patients according to the ethical guidelines approved by the Department of Neurology, School of Medicine, University of São Paulo (0599/10).

**Table 1:** Demographic data from patients analyzed in this study.

Total of cases	Morphology^a^	Mean age at diagnosis (years)^b^	Gender^c^
22	NN	38±7.6	12 F, 10 M
26	AGII	34±8.1	11 F, 15 M
18	AGIII	35±12.3	7 F, 11 M
86	GBM	54±13.9	28 F, 58 M

a NN, non-neoplastic; AGII, low-grade astrocytoma; AGIII, anaplastic astrocytoma; GBM, glioblastoma.

b Age at diagnosis was calculated from date of birth to date of surgery.

c M, male; F, female.

### Sample Preparation

Total RNA was extracted from frozen tissues (tumor and non-neoplastic) using an RNeasy Mini Kit (Qiagen, Hilden, Germany). Evaluation of RNA concentration and purity were carried out by measuring absorbance at 260 and 280 nm. Ratios of 260/280 measures ranging from 1.8 to 2.0 were considered satisfactory for purity standards. Denaturing agarose gel electrophoresis was used to assess the quality of the samples. A conventional reverse transcription reaction was performed to yield single-stranded cDNA. The first strand of cDNA was synthesized from 1 µg of total RNA previously treated with 1 unit of DNase I (FPLC-pure, GE Healthcare, Uppsala, Sweden) using random and oligo (dT) primers, RNase inhibitor, and SuperScript III reverse transcriptase according to the manufacturer’s recommendations (Life Technologies, Carlsbad, USA). The resulting cDNA was subsequently treated with 1 unit of RNase H (GE Healthcare, Uppsala, Sweden), diluted with TE buffer, and stored at −20°C until later use.

### Quantitative Real Time PCR (qRT-PCR)

The relative expression level of *ID4*, *SOX2*, *SOX4*, *OCT-4*, *NANOG* and *CD133* were analyzed by qRT-PCR, using the SYBR Green approach. Quantitative data were normalized in relation to the geometric mean of three housekeeping genes, suitable for the analysis: hypoxanthine phosphoribosyltransferase (*HPRT*), glucuronidase beta (*GUSB*) and TATA box binding protein (*TBP*), as previously demonstrated by our group [26]. The primers were designed to amplify 80–120 bp amplicons, with a melting temperature of 60°C and were synthesized by IDT (Integrated DNA Technologies, Coralville, USA) as follows (5′ to 3′): *ID4* F: TGAACAAGCAGGGCGACAG, *ID4* R: CCCTCTCTAGTGCTCCTGGCT; *SOX2* F: AAGAGAACACCAATCCCATCCA, *SOX2* R: AGTCCCCCAAAAAGAAGTCCA; *SOX4* F: CAGAAGGGAGGGGGAAACATA, *SOX4* R: GAATCGGCACTAAGGAGTTGGT; *NANOG* F: GCAAGAACTCTCCAACATCCTGA, *NANOG* R: CATTGCTATTCTTCGGCCAGTT; *OCT-4* F: CGTGAAGCTGGAGAAGGAGA, *OCT-4* R: CTTGGCAAATTGCTCGAGTT; *CD133* F: TCGGAAACTGGCAGATAGCAA, *CD133* R: GTGAACGCCTTGTCCT; *HPRT* F: TGAGGATTTGGAAAGGGTGT, *HPRT* R: GAGCACACAGAGGGCTACAA; *GUSB* F: GAAAATACGTGGTTGGAGAGCTCATT, *GUSB* R: CCGAGTGAAGATCCCCTTTTTA; *TBP* F: AGGATAAGAGAGCCACGAACCA, *TBP* R: CTTGCTGCCAGTCTGGACTGT. The minimum primer concentrations necessary were determined to give the lowest threshold cycle (Ct) and maximum amplification efficiency, while minimizing non-specific amplification. Primer concentrations used were 150 nM for *ID4*, 200 nM for *HPRT*, *TBP*, *SOX2*, *SOX4* and *OCT-4*, and 400 nM for *GUSB*, *NANOG* and *CD133*. Standard curve was established to ensure amplification efficiency and analysis of melting curves demonstrated a single peak for all PCR products. Additionally, agarose gel electrophoresis was employed to check the size of the PCR product amplified. SYBR Green I amplification mixtures (12 µl) contained 3 µl of cDNA, 6 µl of 2X Power SYBR Green I Master Mix (Life Technologies, Carlsbad, USA) and forward and reverse primers. PCR reactions were run on an ABI Prism 7500 sequence detector (Life Technologies, Carlsbad, USA) as follows: 2 min at 50°C, 10 min of polymerase activation at 95°C, and 40 cycles of 15 s at 95°C and 1 min at 60°C. All the reactions were performed in duplicate. The following equations were applied to calculate gene relative expression according to primer efficiency (E) in tumor samples *versus* the mean of non-neoplastic tissues: 2^−ΔΔCt^ [27] for *SOX2*, *SOX4*, *OCT-4* and *CD133*; and 1+E^−ΔΔCt^ [28] for *ID4* and *NANOG*, where ΔCt = Ct specific gene- geometric mean Ct of housekeeping genes and ΔΔCt = ΔCt tumor – mean ΔCt non-neoplastic. For statistical analysis, gene expression status was scored as high or low expression in relation to the median relative expression value at each grade of astrocytoma.

### DNA Extraction and *TP53* Mutational Analyses

DNA extraction was performed from frozen tumor tissues using All Prep DNA/RNA Mini Kit (Qiagen, Hilden, Germany), and peripheral leukocyte DNA was extracted by a salting-out procedure [29].

Whole coding *TP53* exons (2 to 11) analysis was performed using the polymerase chain reaction single-strand conformation polymorphism (PCR-SSCP) assay and DNA sequencing, as previously reported [30], [31].

### Immunohistochemistry

For immunohistochemical detection, tissue sections were routinely processed and subjected to antigen retrieval. Briefly, slides were immersed in 10 mM citrate buffer, pH 6.0 and incubated at 122°C for 3 min using an electric pressure cooker (BioCare Medical, Walnut Creek, USA). Specimens were then blocked and further incubated with the following antibodies raised against human ID4 (rabbit polyclonal, ab20988, Abcam, Cambridge, UK, 1∶100), SOX2 (mouse clone 6, S1451, Sigma Aldrich, St. Louis, USA, 1∶100), SOX4 (rabbit polyclonal, S7318, Sigma Aldrich, St. Louis, USA, 1∶800) at 16–20°C for 16 hours. Development of the reaction was performed with a commercial kit (Novolink; Novocastra, Newcastle-upon-Tyne, UK) at room temperature, using diaminobenzidine and Harris hematoxylin for nuclear staining. Optimization using positive controls suggested by the manufacturer of each antibody (breast carcinoma for ID4 and SOX4 antibodies, and normal esophagus for SOX2), was performed in order to obtain optimal dilution. Staining intensity of tissue sections was evaluated independently by two observers (SKNM and TFAG). A semi-quantitative score system considering both intensity of staining and percentage of cells was applied as follows: for intensity of staining, 0 = negative, 1 = weak, 2 = moderate and 3 = strong; for cell percentage, 0 = no cells stained, 1 = 10–25%, 2 = 26–50%, 3 = 51–75% and 4 = 76–100%. Only cases with positive cell staining with scores ≥2 were considered as positive. Digital photomicrographs of representative fields were captured and processed using PICASA 3 (Google, Mountain View, USA).

### Statistical Analysis

The statistical analysis of relative gene expression in different grades of astrocytoma was assessed using the Kolmogorov-Smirnov normality test, and the non-parametric Kruskal-Wallis and Dunn tests. Correlation between relative gene expression values was assessed using the non-parametric Spearman-rho correlation test and the parametric Pearson’s correlation test. The Mann-Whitney test was used to compare *TP53* mutational status and relative gene expression. The Kaplan-Meier survival curve was analyzed using the *log-rank* (Mantel Cox) test and multivariate analysis using the Cox proportional hazards model. The logistic regression model included the following parameters: age at diagnosis, gender (female *versus* male), degree of tumor surgical resection (gross total resection (GTR) *versus* partial resection (PR) and gene expression status (hyper or hypoexpression). Differences were considered statistically significant when *p*<0.05. Calculations were performed using SPSS, version 15.0 (IBM, Armonk, USA).

## Results

### Relative Expression Levels in Diffusely Infiltrative Astrocytomas

**Figure 1:**
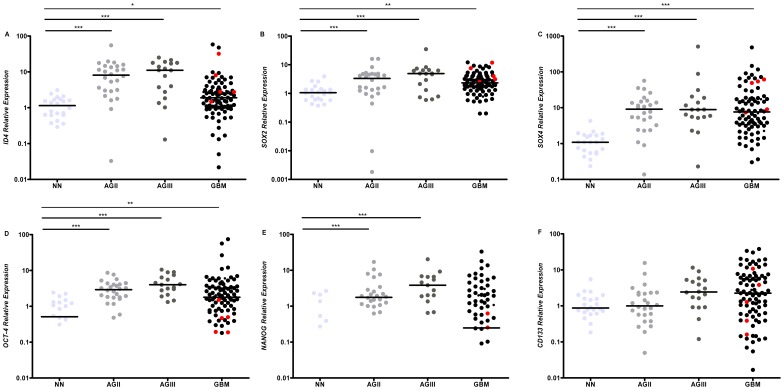
Expression levels of genes in diffusely infiltrative astrocytomas (AGII to GBM). Transcript levels of *ID4* (A), *SOX2* (B), *SOX4* (C), *OCT-4* (D), *NANOG* (E) and *CD133* (F) were determined in 26 low-grade astrocytomas (AGII), 18 anaplastic astrocytomas (AGIII) and 86 GBM cases relative to 22 non-neoplastic (NN) by quantitative real-time PCR. Relative expression values were calculated based on the geometric mean of *HPRT*, *GUSB* and *TBP* expression levels of each sample and non-neoplastic brain values. The following equations were applied to calculate gene relative expression according to primer efficiency (E) in tumor samples *versus* the mean of non-neoplastic tissues: 2^−ΔΔCt^ [27] for *SOX2*, *SOX4*, *OCT-4* and *CD133*; and 1+E^−ΔΔCt^ [28] for *ID4* and *NANOG*, where ΔCt = Ct specific gene – mean Ct of housekeeping genes and ΔΔCt = ΔCt tumor – mean ΔCt non-neoplastic. Red dots represent the secondary GBM cases. Horizontal bars show the median of each group and the values are presented in Table 2. *NANOG* expression in 15 NN and 40 GBM cases was very low and, as a result, the horizontal bar for NN does not appear in the graphic (median = 0). The difference of relative gene expressions among the groups were statistically significant (*p*<0.0005 for *ID4*, *SOX2*, *SOX4*, *OCT-4* and *NANOG*; and *p*<0.05 for *CD133,* Kruskal-Wallis test). A pair-based comparison was assessed using Dunn test. The *p* value results are shown, where ***p<0.0005, **p<0.005 and *p<0.05.

**Figure 2:**
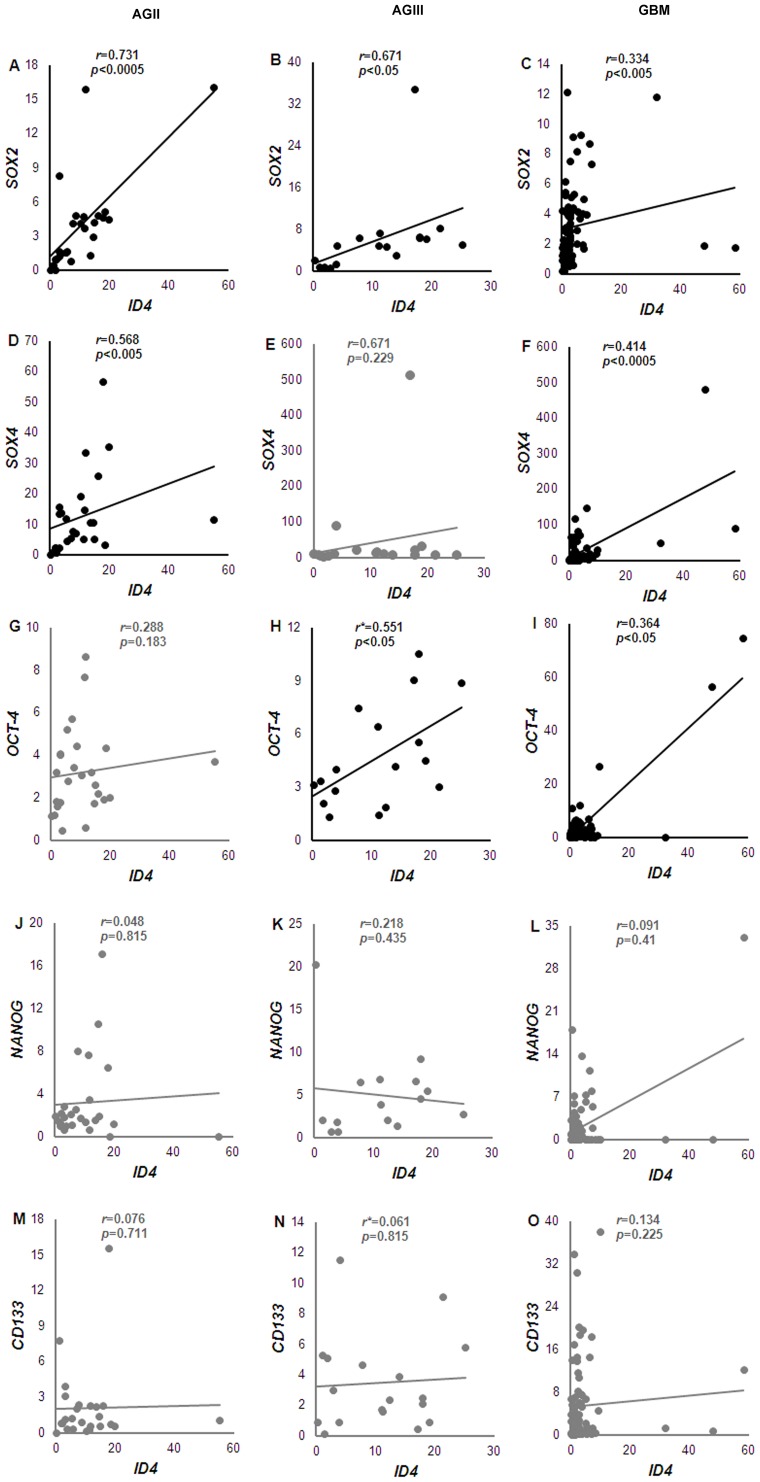
Correlation between *ID4* and *SOX2*, *SOX4*, *OCT-4*, *NANOG* and *CD133* expression levels in diffusely infiltrative astrocytomas. Correlation was assessed in AGII (A, D, G, J, M), AGIII (B, E, H, K, N) and GBM (C, F, I, L, O) cases. *ID4* expression level was correlated to *SOX2* (A-C), *SOX4* (D-F), *OCT-4* (G-I), *NANOG* (J-L) and *CD133* (M-O) expression levels. The significant correlations are shown in black and the non-significant in grey. *r* correlation coefficient assessed by Spearman-rho test, and r* by Pearson’s correlation test.

Gene expression analysis by qRT-PCR for *ID4* showed higher median expression levels in all diffusely infiltrative astrocytoma cases (AGII to GBM) relative to the NN cases, and comparison among the groups was statistically significant (Figure 1A, *p*<0.0005, Kruskall-Wallis test). Although the *ID4* median expression level in GBM cases was lower than in AGII and AGIII, there was a variability of these expression values, with cases presenting both higher and lower values than the other grades. Similar variability of *ID4* expression was also observed in AGII and AGIII (Figure 1A). A multivariate Cox regression model (which considered age at diagnosis, gender, degree of tumor surgical resection, and *ID4* expression status) showed that *ID4* expression (hyper or hypoexpression) alone had no impact on patient’s prognosis. Only age at diagnosis was an independent prognostic factor (hazard ratio = 1.02, *p* = 0.02). *SOX2*, *SOX4*, *OCT-4*, *NANOG*, and *CD133* also showed higher mRNA levels in AGII-GBM cases in comparison to NN, as shown in Figure 1B–1F. *SOX2* expression levels were compared to *ID4* levels to verify the degree of their co-expression in human diffusively infiltrative astrocytomas. Interestingly, the correlation analysis of *SOX2* showed mRNA levels similar to *ID4*, with positive correlation found in AGII (*r* = 0.731; *p* = 0.00002), AGIII (*r* = 0.671; *p* = 0.006) and GBM (*r* = 0.334; *p* = 0.0006). Next, *SOX4, OCT-4*, *NANOG* and *CD133* expression levels were also evaluated. *SOX4* expression levels were similar to those of *ID4*, although positive correlation was only found in AGII (*r* = 0.568; *p* = 0.002) and GBM (*r* = 0.414; *p* = 0.00009). *OCT-4* relative expression correlated positively with *ID4* in AGIII (*r* = 0.551; *p* = 0.02) and GBM (*r* = 0.364; *p* = 0.01). In contrast to the other analyzed genes, several GBM cases exhibited very low expression levels of *NANOG*, and no correlation was found between *ID4* and *NANOG* expression levels. *ID4* and *CD133* expressions did neither not correlate. An overview of the results of analyzed correlations is shown in Figure 2.

**Table 2:** Median of relative expression levels of the analyzed genes in astrocytomas, according to morphology.

Morphology^a^	*ID4*	*SOX2*	*SOX4*	*OCT-4*	*NANOG*	*CD133*
NN	1.15	1.06	1.09	0.51	0	0.87
AGII	8.12	3.32	9.12	2.92	1.77	1
AGIII	11.1	4.9	8.86	4	3.84	2.43
GBM	1.89	2.32	7.63	1.79	0.25	2.26

a NN, non-neoplastic; AGII, low-grade astrocytoma; AGIII, anaplastic astrocytoma; GBM, glioblastoma.

It is interesting to note that secondary GBM cases (red dots on Figure 1) exhibited a higher median expression level for *ID4* (2.78) than did primary GBM cases (1.84). Similar results were obtained for *SOX2* (3.96 for secondary and 2.26 for primary GBM) and *SOX4* (48.99 for secondary and 6.74 for primary). In contrast, the median of OCT-4 expression was 0.47 in secondary GBM and 2.03 in primary GBM; the median of *NANOG* expression level in secondary GBM was 0.13 while 0.35 in primary GBM, and the median of *CD133* expression level was 1.28 for secondary GBM and 2.26 for primary GBM. To further investigate the factors contributing to these differences, the expression values were analyzed according to *TP53* mutation status.

### Association between *ID4*, *SOX2*, *SOX4*, and *NANOG* mRNA Expressions and *TP53* Mutation Status

**Figure 3:**
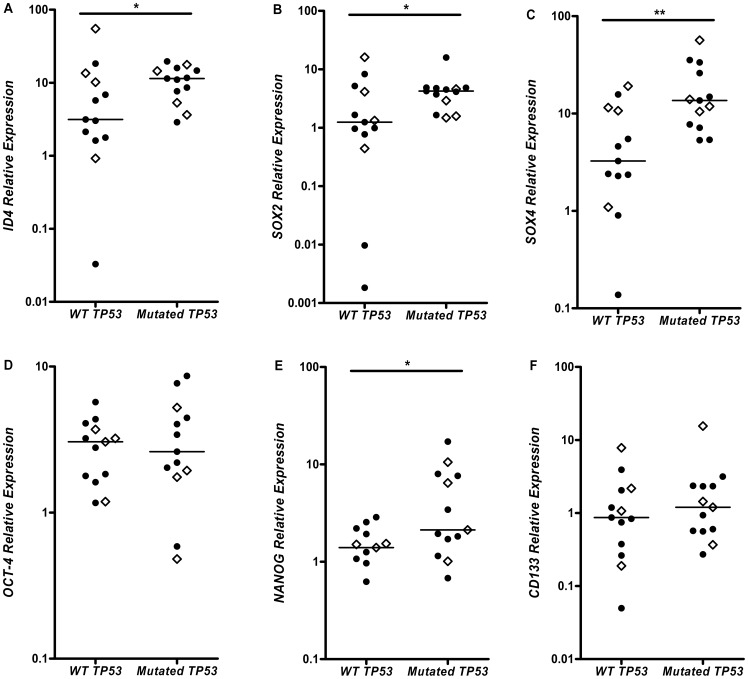
Comparison of gene expression levels between the wild-type *TP53* (*WT TP53*) and the mutated *TP53* (*Mutated TP53*) in AGII cases. Higher expressions of *ID4* (A), *SOX2* (B), *SOX4* (C) and *NANOG* (E) were observed on the mutated *TP53* AGII cases. No difference was found for *OCT-4* (D) and *CD133* (F) relative expression between the two groups. White lozenges represent the deceased patients. The *p* values are: *p<0.05 and ** *p*<0.005, Mann-Whitney test.

The frequency of *TP53* mutation was 11.6% in GBM (10 out of 86), 16.6% in AGIII (3 out of 18) and 50% in AGII (13 out of 26), as described in our previous studies [30], [31] (Table S1). Our GBM series is composed mainly by primary GBMs, which explains the low frequency of *TP53* mutation and corroborates the classification based on clinical presentation. The low frequency of *TP53* mutations in GBM and AGIII cases did not permit statistical analyses of the proposed parameters; however, this analysis was feasible among AGII cases. Interestingly, *TP53*-mutated AGII cases showed higher relative expression of *ID4* when compared to AGII cases with wild-type *TP53* (*p* = 0.048) (Figure 3A). Also, *SOX2* (*p* = 0.044), *SOX4* (*p* = 0.004) and *NANOG* (*p* = 0.025) relative expressions were higher in mutated than in wild-type *TP53* in AGII cases (Figure 3B, 3C and 3E respectively). No difference was found for *OCT-4* relative expression between wild-type and mutated *TP53* cases (Figure 3D). Despite the fact that *TP53-*mutated AGII cases displayed slightly higher relative expression of *CD133*, the difference was not statistically significant (Figure 3F). No difference in expression was found regarding the different types of *TP53* mutations (whether missense, nonsense or in splicing sites). Mann-Whitney test was applied for all the above statistical analysis. Moreover, no significant impact was observed in the overall survival time or in the progression free survival time in AGII cases, concerning either relative expression levels of *ID4, SOX2, SOX4, OCT4, NANOG, and CD133* or *TP53* mutational status (results presented Table S2). Although *TP53* mutated AGII cases presented a median of 40 months of overall survival time compared to a median of 51 months of wild-type *TP53* AGII cases, it did not reach statistical significance because of the small number of cases in each group (Figure 3, white lozenges for deceased AGII patients).

**Figure 4:**
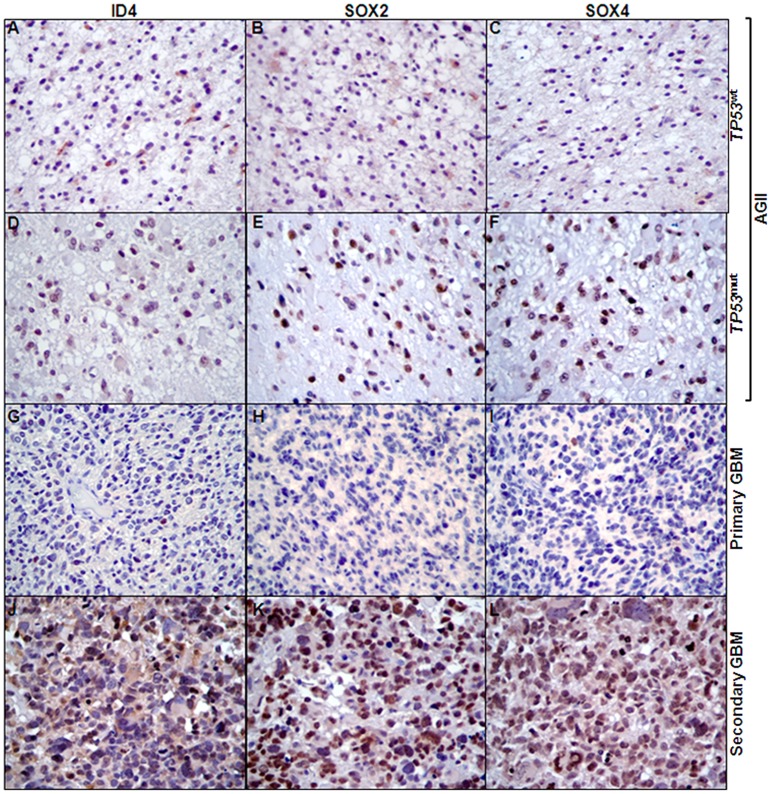
ID4, SOX2 and SOX4 immunohistochemistry. Representative cases of wild-type *TP53* AGII (A-C), mutated *TP53* AGII (D-F), primary GBM (G-I) and secondary GBM (J-L) stained for ID4, SOX2 and SOX4 are demonstrated. Both mutated AGII and secondary GBM cases showed stronger and larger number of nuclear stained cells (score 3 for intensity and ≥75% of positive cells) for ID4, SOX2 and SOX4. Comparatively, wild-type *TP53* AGII and primary GBM presented score 1 for intensity and <25% of positive cells. The reaction was performed in paraffin embeded tissue sections with a commercial polymer kit (Novolink; Novocastra, UK), using diaminobenzidine as developer and Harris hematoxylin for nuclear counterstaining. 200× magnification for all images.

Associated expression of *ID4*, *SOX2* and *SOX4* with *TP53* mutational status was further confirmed at the protein level by immunohistochemistry. The wild type *TP53* AGII cases (Figure 4A–4C) showed weak or no staining for the three targets in comparison to the *TP53*-mutated cases (Figure 4D–4F), as did the primary GBM cases (Figure 4G–4I) when compared to the secondary GBM (Figure 4J–4L) cases.

**Figure 5:**
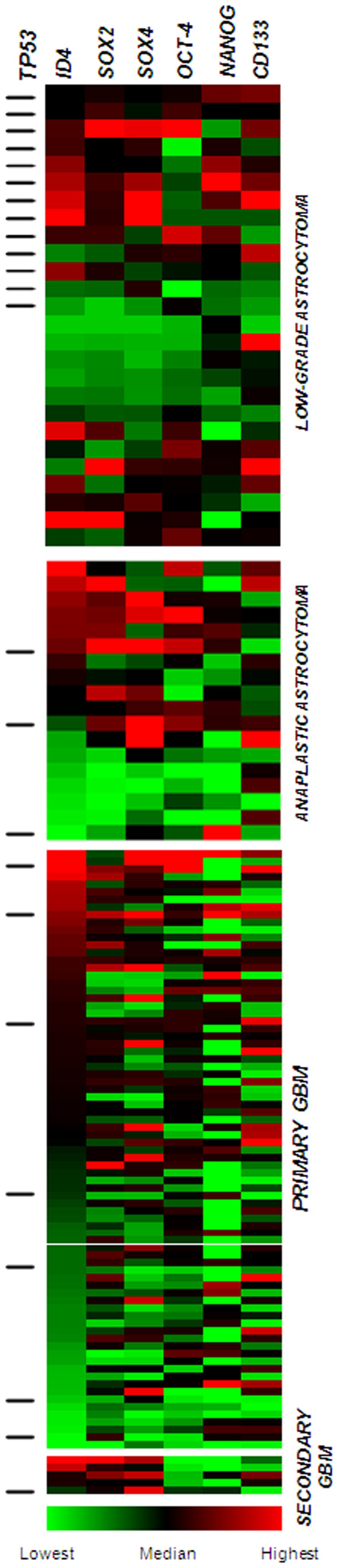
Heatmap displaying the relative gene expressions in low-grade astrocytoma (AGII), anaplastic astrocytoma (AGIII) and GBM cases according to *TP53* mutation status. The *TP53* mutated cases are represented by side dashes. The mutated *TP53* AGII cases showed more elevated expression levels of *ID4*, *SOX2*, *SOX4* and *NANOG*. *CD133* expressions were more heterogeneous among the cases. *SOX2* and *SOX4* showed similar expression levels to *ID4*. Similarly, secondary GBM cases also presented higher *ID4*, *SOX2*, *SOX4* expression levels. *OCT-4*, *NANOG* and *CD133* expression levels were heterogeneous among secondary GBM cases, and *OCT-4* presented higher mRNA levels in primary GBM.

The overview of *TP53* mutation status, relative gene expression for AGII, and expression differences among AGII, AGIII, primary and secondary GBM are displayed as heatmap in Figure 5.

### Impact of *ID4*, *SOX4* and *OCT-4* Expression Levels on Clinical Outcome for GBM Patients

**Figure 6:**
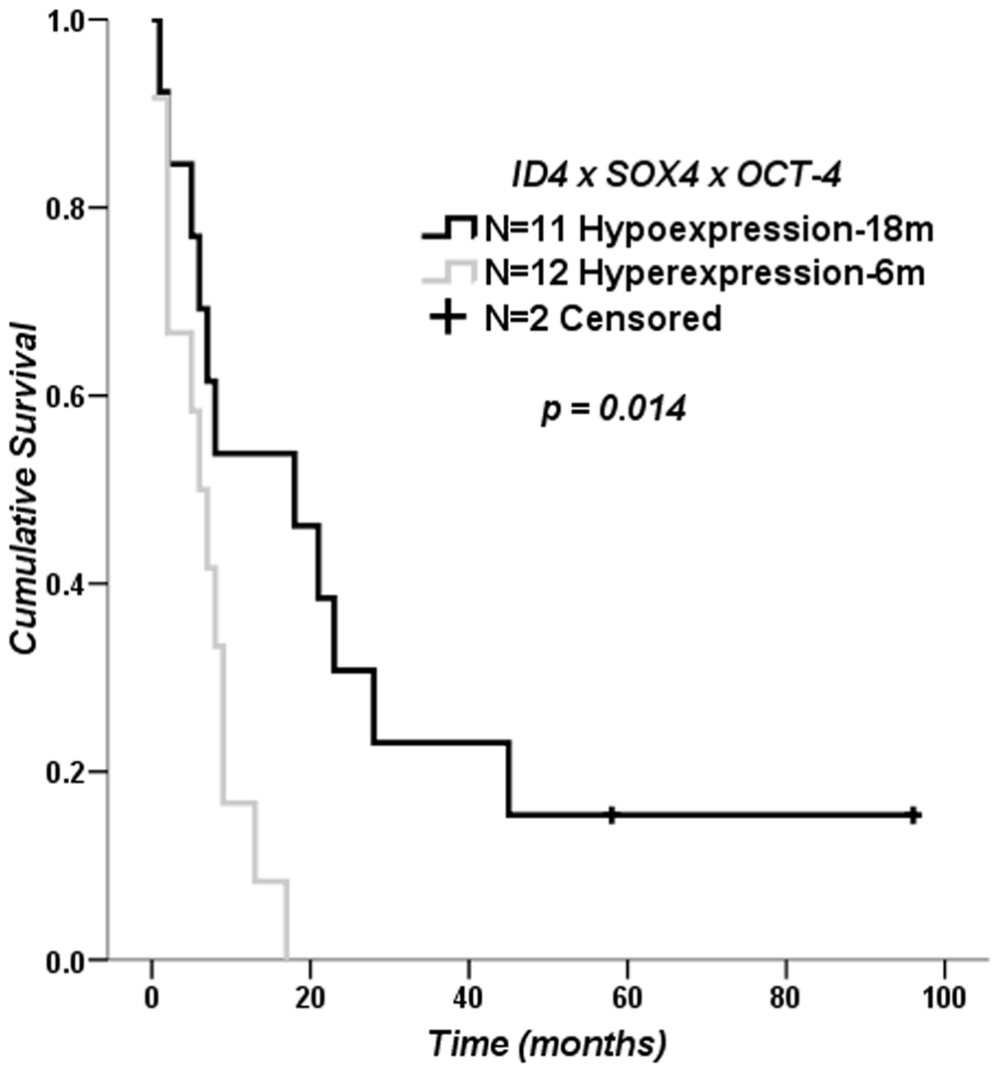
Survival curve of GBM patients. Twenty-five GBM cases out of 86 GBM cases presented concomitant high or low *ID4*, *SOX4* and *OCT-4* relative expresssion levels (12 GBM cases presenting high expressions and 13 low expressions for the three genes). The survival time difference between the two groups was statistically significant (*log rank p*-value = 0.014), presenting median survival time of 6 months for GBM cases presenting concomitant high expressions for the three genes compared to 18 months for GBM cases with low expressions.

Considering the variability of the relative expression values found in GBM cases, we evaluated the impact of up-regulation of the analyzed genes on overall patient survival. For the evaluation, conditions were determined for high and low gene expression. Secondary GBM cases were excluded from this analysis due to the small number of cases. None of the genes had an impact on overall survival, either on their own or when grouped in pairs for the determined conditions (Figure S1). However, there was a significant difference when comparing GBM cases with high *ID4*, *SOX4* and *OCT-4* expressions (median survival of 6 months) with cases with low expressions for the three genes (median survival of 18 months) (*log rank p* = 0.014), as shown on the Kaplan-Meier survival curve in Figure 6.

## Discussion

We have demonstrated a differential expression of *ID4* in human diffusely infiltrative astrocytoma cases demonstrating association with *TP53* mutation status, as well as to *SOX2*, *SOX4* and *OCT-4* mRNA expression levels.

### *ID4* mRNA Levels are Elevated in Astrocytomas in Comparison to Non-neoplastic Brain Tissue

Our study demonstrated significantly higher mRNA expression levels of *ID4* in astrocytomas when compared to non-neoplastic brain tissue. Similar results have also been described for ID1–3 proteins in astrocytomas, with higher expression levels in tumors than in non-neoplastic white-matter [32]. A previous immunohistochemical report has shown stronger ID4 expression in GBM compared to AGII, AGIII and normal brain tissue [12]. Such association was not significant in our study, most probably due to a larger number of cases analyzed herein, and also to the heterogeneity inherent to GBM, here corroborated by the widespread *ID4* mRNA expression among the studied GBM cases. Nevertheless, the increased *ID4* expression level in diffusely infiltrative astrocytoma is in accord with the tumor re-expression model of IDs [4], postulating *ID4* as an additional marker of astrocytoma progression in malignancy.

A recent report [34] has shown that *ID4* promoter methylation was an independent factor on patient’s prognosis, and that association of *ID4* promoter methylation and *MGMT* methylation status conferred significantly longer overall survival to GBM patients. We assessed the correlation between *ID4* hypoexpression and the *MGMT* methylation status in GBM, previously reported by our group [33]. The Cox regression model showed only *MGMT* status as an independent factor for prognosis (hazard ratio = 4.684, *p* = 0.014), differing from the previous report. Further studies on *ID4* promoter methylation are needed in the present GBM series.

### *ID4* Hyperexpression is Driven by Mutated *TP53* in AGII

Here we demonstrate a significant difference in *ID4* expression between AGII cases harboring *TP53* mutation versus wild-type, mutated cases showing significant increase in *ID4* expression. Other studies in breast cancer models have demonstrated *in vitro ID4* up-regulation driven by the mutated p53 protein [13], [14]. *TP53* mutations, present in 50% of AGII cases, are considered one of the earliest events in astrocytoma formation [9]. The significant association shown here between *TP53* mutation and *ID4* expression could possibly classify *ID4* hyperexpression as an early event in astrocytoma formation. The analysis of AGII patients OS time in *TP53* mutated and wild-type cases showed that the mutated cases had a shorter survival by eleven months in comparison to the wild-type group. Considering the low number of cases, further studies are necessary to confirm statistically this result. The great majority of *TP53* mutations are missense, localized in specific gene domains (“*hot spots*”) that do not inactivate protein function. On the contrary, these alterations stabilize the mutated protein and enhance its oncogenic activity by increasing the transcription of target genes, recruiting other transcription factors and co-factors (recently reviewed in [35]). However, no difference of the analyzed gene expressions were found compared to the different types of *TP53* mutation in the current AGII series, and it remains to be elucidated if the cases harboring inactivating nonsense mutations present alternative activation for *ID4*, *SOX2* and *SOX4*.

### Associated Expression of *ID4* with *SOX2* and *SOX4* and with Mutated *TP53*

*SOX2* also proved to be significantly augmented and correlated to *ID4* in *TP53* mutated AGII cases. *SOX2* overexpression driven by inactivation of p53 in mouse embryonic fibroblasts has been demonstrated [36], although the mechanism remains unknown. One possible explanation is that *ID4* up-regulation activates *SOX2* through inhibition of a microRNA, mir-9*, which is a direct negative regulator of *SOX2*, as shown in glioma cell lines [16]. Our findings of *ID4* up-regulation associated to *TP53* mutated status and to *SOX2* hyperexpression in human astrocytoma specimens corroborate these previous observations in cell lines. Taken together, these data suggest that *ID4* and *SOX2* act jointly post-*TP53* mutation in promoting astrocytoma tumorigenesis.

The association between SOX4 and p53 has also been reported [37], [38], with SOX4 stabilizing p53 protein and inhibiting its induction of the apoptotic pathway. In our study, *SOX4* expression was increased in *TP53* mutated cases, in a similar pattern to *ID4*. It remains to be elucidated what role the observed association between *SOX4* and mutated *TP53* plays in the process of astrocytic tumor formation.

Our results showed a significant increase in *NANOG* expression in *TP53* mutated AGII cases. It is known that p53 is a direct negative regulator of *NANOG* [39] and that the absence of a functional p53 protein augments *NANOG* expression. *NANOG* levels did not correlate to any of the other analyzed targets, and its expression pattern in GBM cases was random, enabling us to speculate that *NANOG* works differently to contribute to astrocytoma formation. *CD133* levels were not significantly different when *TP53* mutated and wild-type AGII cases were compared, and GBM cases also displayed a random pattern, suggesting that *CD133* also works differently in the tumorigenic process of astrocytomas.

*OCT-4* relative expression was not influenced by *TP53* mutational status and did not correlate with *ID4* expression in AGII cases. However, the expression pattern of GBM cases was strikingly different: secondary GBM exhibited very low *OCT-4* mRNA levels in comparison to primary GBM. Together with the positive correlation between *OCT-4* and *ID4* found in both AGIII and GBM cases, these data indicate a role for this target in the most malignant grades of astrocytoma. These results prompted us to further investigate the combined expression of *ID4*, *SOX4* and *OCT-4*.

### Impact of *ID4*, *SOX4* and *OCT-4* Mutual Hyperexpression on Primary GBM Patients’ Overall Survival

The expression level variability among GBM cases was present for all analyzed genes (Figure 1), with some cases exhibiting very high mRNA levels in contrast to low levels found in others. Again, this phenomenon may be due to the extensive heterogeneity found in GBM at both the cellular and molecular levels [40], contributing to difficulties in eradicating these tumors. Thus, we believed it was necessary to ascertain whether patients bearing tumors with higher mRNA levels of the analyzed genes showed worse overall survival. When we grouped *ID4*, *SOX4* and *OCT-4* together, patients hyperexpressing these genes exhibited much lower survival time. In bladder cancers, both *ID4* and *SOX4* were amplified and overexpressed heterogeneously [41], similar to astrocytomas, and contributed to the variable biological and clinical behavior of the tumors. As previously mentioned, OCT-4 and SOX4 proteins form a transcription complex and induce *SOX2* expression, increasing the tumorigenicity of glioma cells. Although decreased survival was demonstrated in mice inoculated with GBM cells hyperexpressing *OCT-4*, we observed that *OCT-4* alone had no impact on our patients’ overall survival (Figure S1D). Because ID4 alone, as well as the SOX4 and OCT-4 complex, activates *SOX2*, and because chemoresistance is associated with both *SOX2* and *ID4* augmented expression, it is possible to speculate that multiple *SOX2* activation events in GBM may impair patient prognosis. SOX2 and OCT-4, are considered masters of pluripotency in embryonic stem cells [42]. This role is maintained in cancer stem cells (CSC), a subset of tumor cells regarded as possessing traits such as therapeutic resistance, tumor angiogenesis and recurrence [43]. ID4 has also shown to play an important role in CSC biology, its expression being imperative to the formation and maintenance of CSC population [44]. In GBM stem cells [45]–[48], ID4 has been postulated as an important target in the dedifferentiation process, as shown in the *in vitro* reports [15], [16]. Moreover, the re-expression of embryonic stem cells genes in tumors, including gliomas, has been associated with a more aggressive phenotype [22], [49], [50]. It is possible that this is the cause for the worse clinical end-point of overall survival among GBM patients found in our study. However, because of the low number of GBM cases (n = 25) in which this finding was demonstrated, the present result should be validated in an independent study sample containing a higher number of GBM patients.

In this scenario, *ID4* seems to be a promising target for further studies in order to better understand its role in tumorigenesis and its potential use in therapeutics.

## Supporting Information

Figure S1 Kapplan-Meier curves of GBM patients according to relative expression levels of *ID4*, *SOX2*, *SOX4*, *OCT-4*, *ID4*x*SOX2*, *ID4*x*SOX4*, *ID4*x*OCT-4*, *SOX4*x*OCT-4* (TIF).
(TIF)

Table S1 Clinical data of patients in the study (Excel).
(XLS)

Table S2 Low-grade astrocytoma patients’ survival time analysis (Word).
(DOC)
