# Supplementary material for: Differential Expression of ID4 and Its Association with TP53 Mutation, SOX2, SOX4 and OCT-4 Expression Levels
Source: PLoS One. 2013 Apr 16;8(4):e61605. doi: 10.1371/journal.pone.0061605 (PMC3628974; doi:10.1371/journal.pone.0061605)
Supplement: Table S2 — Low-grade astrocytoma patients’ survival time analysis (Word). (DOC) [file pone.0061605.s003.doc]

| **Table S2: Low-grade astrocytoma patients’ survival time analysis** | | | | |
| --- | --- | --- | --- | --- |
|  | **Deceased** | | **Alive** | |
|  | **n/OS**^a^ **(months)** | | **n/PFS**^b^ **(months)** | |
|  | 3/51 | 4/40 | 9/44 | 7/32 |
|  | **Median of relative expression level** | | | |
|  | ***TP53^wt^*** ^c^ | ***TP53^mut^*** ^d^ | ***TP53^wt^*** ^c^ | ***TP53^mut^*** ^d^ |
| ***ID4*** | 10.18 | 9.9 | 3.02 | 11.66 |
| ***SOX2*** | 4.13 | 2.25 | 0.99 | 4.5 |
| ***SOX4*** | 11.5 | 12.91 | 2.4 | 13.61 |
| ***OCT-4*** | 3.05 | 1.85 | 2.78 | 3.42 |
| ***NANOG*** | 1.4 | 4.29 | 1.26 | 1.82 |
| ***CD133*** | 1.07 | 1.32 | 0.83 | 2.32 |
| **Total (n)**^e^ **= 23** | | | | |
| ^a^OS, overall survival time | | | | |
| ^b^PFS, progression free survival time | | | | |
| ^c^*TP53^wt^*, wild-type *TP53* | | | | |
| ^d^*TP53^mut^*, mutated *TP53* | | | | |
| ^e^Three recurrent AGII cases were excluded from the analysis | | | | |
| Clinical and follow-up data available at [1] | | | | |

**Figure 1 in Supporting Information**

**Figure S1:** **Kapplan-Meier curves of GBM patients according to relative expression levels of *ID4*, *SOX2*, *SOX4*, *OCT-4*, *ID4*x*SOX2*, *ID4*x*SOX4*, *ID4*x*OCT-4*, *SOX4*x*OCT-4*.** A) Analysis of *ID4* relative expression levels in 80 GBM samples (39 higher expression, 1 excluded/censored of the analysis as patient is still alive; and 37 lower expressions, 3 excluded/censored). No difference was found between groups (median survival 8 and 7 months respectively, *p*=0.325). B) Analysis of *SOX2* relative expression levels in 80 GBM samples (37 higher expression, 3 excluded/censored; and 37 lower expressions, 1 excluded/censored). No difference was found between groups (median survival 8 and 7 months respectively, *p*=0.891). C) Analysis of *SOX4* relative expression levels in 80 GBM samples (39 higher expression, 2 excluded/censored; and 37 lower expressions, 2 excluded/censored). No difference was found between groups (median survival 8 months in both groups, *p*=0.376). D) Analysis of *OCT-4* relative expression levels in 80 GBM samples (40 higher expression, 1 excluded/censored; and 36 lower expressions, 3 excluded/censored). No difference was found between groups (median survival 8 months in both groups, *p*=0.218). E) Analysis of *ID4* and *SOX2* relative expression levels in 54 GBM samples (26 higher expression, 1 excluded/censored; and 26 lower expressions, 1 excluded/censored). No difference was found between groups (median survival 8 months in both groups, *p*=0.639). F) Analysis of *ID4* and *SOX4* relative expression levels in 48 GBM samples (24 higher expression, one excluded/censored; and 24 lower expressions, 2 excluded/censored). No difference was found between groups (median survival 8 months in both groups, *p*=0.336). G) Analysis of *ID4* and *OCT4* relative expression levels in 43 GBM samples (22 higher expression and 19 lower expressions, 2 excluded/censored). No difference was found between groups (median survival 7 months in both groups, *p*=0.146). H) Analysis of *SOX4* and *OCT-4* relative expression levels in 38 samples (20 higher expression, one excluded/censored; and 18 lower expressions, 2 excluded/censored). No difference was found between groups (median survival 7 and 8 months respectively, *p*=0.170).

References

1. Bianco A de M, Miura FK, Clara C, Almeida JRW, Silva CC da, et al. (2013) Low-grade astrocytoma: surgical outcomes in eloquent versus non-eloquent brain areas. Arq Neuropsiquiatr 71: 31–34.
